# Identifying phenotypic and physiological subgroups of preschoolers with autism spectrum disorder

**DOI:** 10.1017/S0033291721003172

**Published:** 2023-03

**Authors:** Tessel Bazelmans, Emily J. H. Jones, Sheila Ghods, Sarah Corrigan, Karen Toth, Tony Charman, Sara J. Webb

**Affiliations:** 1 Psychology Department, Institute of Psychiatry, Psychology & Neuroscience, King's College London, London, UK; 2 Centre for Brain and Cognitive Development, Birkbeck, University of London, London, UK; 3 Center for Child Health, Behavior & Development, Seattle Children's Research Institute, Seattle, WA, USA; 4 Department of Psychiatry and Behavioral Science, University of California San Francisco, San Francisco, CA, USA; 5 Department of Psychiatry & Behavioral Sciences, University of Washington, Seattle, WA, USA

**Keywords:** autism spectrum disorder, autonomic activity, heart rate, latent profile analysis, social skills

## Abstract

**Background:**

To understand the emergence of symptoms in autism spectrum disorder (ASD), we need to identify the mechanisms that underpin the development of core social skills. Mounting evidence indicates that young children with later ASD attend less to other people, which could compromise learning opportunities with cascading effects. Passive looking behaviour does not tell us about engagement with visual information, but measures of physiological arousal can provide information on the depth of engagement. In the current study, we use heart rate (HR) and heart rate variability (HRV) to measure engagement with social dynamic stimuli in ASD.

**Methods:**

Sixty-seven preschoolers with ASD and 65 typical developing preschoolers between 2 and 4 years of age participated in a study where HR was measured during viewing of social and non-social videos. Using latent profile analyses, more homogeneous subgroups of children were created based on phenotype and physiology.

**Results:**

Preschool-aged children with ASD, regardless of their non-verbal, verbal and social competencies, do not differ in overall HR or HRV compared to TD children. However, the ASD group showed a larger increase in HR (more disengagement) than the TD group to later-presented social stimuli. Phenotypic and physiological profiles showed this was primarily the case for children with below average verbal and non-verbal skills, but not necessarily those with more ASD symptoms.

**Conclusion:**

Children with ASD, especially a subgroup showing moderate cognitive delays, show an increase in HR to social stimuli over time; this may reflect difficulties re-engaging with social information when attention is waning.

Autism spectrum disorder (ASD) is characterised by difficulties in social interaction and communication and the presence of restricted or repetitive behaviours (American Psychiatric Association, 2013) and often co-occurs with language difficulties (Kwok, Brown, Smyth, & Oram Cardy, 2015). Reliable diagnosis of ASD is possible from 24 months (Ozonoff et al., 2015), but often does not occur in the community until childhood or later (Brett, Warnell, McConachie, & Parr, 2016). Identifying robust objective markers of early-emerging social or communication atypicalities may improve this picture. A promising domain is social attention, for which there is mounting evidence of disruption in early ASD (Chita-Tegmark, 2016; Klin, Shultz, & Jones, 2015). Key observations include reduced social orienting, joint attention (Dawson et al., 2004) and attention to eyes (Klin, Jones, Schultz, Volkmar, & Cohen, 2002). Alterations in early attention patterns may have consequences for developing communication skills: for example, social orienting and joint attention are related to language in 1−4-year olds with and without ASD (Dawson et al., 2004) and attention to a person over an object is associated with parent report of both social and communication skills in 2−6-year olds with ASD (Murias et al., 2018).

Measurements of looking behaviour can provide insight into what children could potentially take in but provides limited insight into how deeply that information is processed (e.g. Richards, 1997*a*, *b*). Previous studies have shown that active engagement ends before visual attention is terminated (Richards, 1997*b*) and the type of (physiological) attention has an effect on how stimuli are processed (Richards, 1997*a*). Disruptions in social engagement in autism are already observed in infancy, both on a cognitive and a neural level (Jones et al., 2016). Thus, the depth with which a stimulus is processed and whether it is prioritised for later learning and memory is dependent on the *engagement state* of the child during viewing.

Measurements of the autonomic nervous system like heart rate (HR) or heart rate variability (HRV) can provide critical information about engagement states during visual attention (Richards & Casey, 1992; Richards & Cronise, 2000; Thayer, Ahs, Fredrikson, Sollers, & Wager, 2012). The autonomic nervous system modulates responses to information and events (Thayer & Lane, 2000), decreasing output in periods of relative calm attention and increasing physiological output in periods of perceived stress. Infants are less easily distracted during periods of slowing HR (Lansink & Richards, 1997), moments of sustained attention when information processing takes place (Frick & Richards, 2001; Richards, 1997*a*). Calm, attentive states signalled by low HR and high HRV may indicate greater receptiveness to social interaction, with less distress or distraction (e.g. de Barbaro, Clackson, & Wass, 2017; Porges, 2009). Thus, physiological responses can provide information about a child's internal state and the degree to which they are likely to learn and remember information they experience.

A recent meta-analysis showed that in addition to lower HRV at baseline, individuals with ASD also show lower HRV and less HRV reactivity to social stress and cognitive tasks compared to controls (Cheng, Huang, & Huang, 2020). In children, studies comparing responses to social (e.g. child-directed speech) and non-social (e.g. toys) dynamic stimuli have reported similar ‘chronic’ patterns of hyper-arousal that may be apparent across a range of contexts (Vaughan Van Hecke et al., 2009; Watson, Roberts, Baranek, Mandulak, & Dalton, 2012). For example, Watson et al. (2012) found that 2−3-year olds with ASD had increased HR, but no differences in HRV, compared to a typical developing (TD) group during social/non-social videos. Vaughan Van Hecke et al. (2009) reported decreased HRV in 8−12-year olds in ASD compared to TD children during social/non-social videos. Additionally, children with ASD showed a larger deceleration in HRV to a video of an unfamiliar person, compared to the TD group. Thus, to date there is evidence for increased arousal (which may reflect increased stress and/or decreased attention engagement) more generally in young children with ASD. Such findings can be contrasted with recent evidence of typical physiological activity in a large group of preschoolers with ASD during a less content-rich presentation (watching wildlife videos; Bazelmans et al., 2019).

Subtyping of children with ASD may be beneficial to understand conflicting findings. Variability in ASD, both phenotypically and biologically, is high and stratification may help in finding significant effects for subtypes of ASD (Loth et al., 2016). Differences in physiological responses are dependent on context and may vary in directionality and magnitude between young children with ASD. Data-driven approaches can help identify whether there are groups of children that respond in a different way to social and non-social stimuli, e.g. based on intellectual or cognitive ability (see Patriquin, Hartwig, Friedman, Porges, & Scarpa, 2019). Thus, looking at the ASD group as a whole might be masking subgroups of children who do show either chronic physiological differences or differences in response to social and non-social information.

## Aim of the study

In the current study, we tested whether preschoolers with ASD show a different physiological response to social *v.* non-social videos compared to TD preschoolers. We compared groups using (1) a ‘case−control’ approach and latent classes based on (2) phenotypic and (3) physiological profiles. Based on the findings of chronic hyperarousal in children with ASD and previous associations between arousal and both language and social skills to various stimuli (Neuhaus, Bernier, & Beauchaine, 2014; Patriquin, Scarpa, Friedman, & Porges, 2013; Watson, Baranek, Roberts, David, & Perryman, 2010), we expected that there would be a phenotypic class of children with increased arousal during both social and non-social videos and that those showing greatest phenotypic impairment would show least stimulus-related flexibility in autonomic response (Patriquin et al., 2019). Considering that we did not find hyperarousal during calm, non-social, animal videos in children with ASD (Bazelmans et al., 2019), we were interested to see if there are children who show an increase to arousal to social stimuli specifically. We tested the same hypotheses as above using physiological profiles to see if classes with meaningful and potentially more specific phenotypic profiles could be identified based on physiology and compared these to the phenotypic classes. Taken together, we examined if there was converging evidence of an association between social physiological arousal and developmental level, social skills and ASD symptoms.

## Methods

### Participants

Participants were recruited from clinical and community sources; guardian/parents provided informed consent. In all, 110 ASD and 88 TD preschool children between 2 and 4 years were enrolled in a study of attention and emotion regulation (see Bazelmans et al., 2019). Children had English as their primary language and did not have serious medical or neurological conditions, motor impairments (>70 on the Vineland Adaptive Behaviour Scale-II; VABS), vision or hearing loss, history of severe ear infections, or birth weight below 1500 g and a gestational age below 34 weeks.

Preschoolers in the ASD group met criteria for ASD on the Autism Diagnostic Observation Schedule (ADOS) and DSM-IV criteria (American Psychiatric Association, 2000) based on all available information. Children in the TD group were included if they additionally had no history of parent or pediatrician concerns regarding the child's development, all VABS domain standard scores ⩾80, and did not have a first-, second- or third-degree relative with ASD.

#### Final sample

The final sample consisted of 131 children between 24 and 59 months, 66 (6 females) with ASD and 65 (11 females; χ^2^(1) = 1.78, *p* = 0.182) TD. Forty-four children (24 ASD and 20 TD) withdrew or were excluded after the first timepoint based on additional medical information that might impact HR measurement (e.g. medication taken during pregnancy with potential impact on cardiovascular development) or inconsistent or missing clinical information (e.g. ADOS score) to confirm their ASD or TD status. One additional child in the ASD group was excluded from the analysis because of missing data on visual reception and was identified as an outlier on HR. Twenty-two children (19 ASD and 3 TD) were excluded because of no or not enough valid HR data (see ‘Autonomic measures’). Characteristics of the included *v.* excluded children in the ASD group did not differ (all *p*s ⩾0.661; see Supplementary Material (SM) 1.1). Fifty children with ASD and 52 TD children had HR data available at both sessions.

Children in the ASD group scored lower on visual reception, language, communication and socialisation (all *p* < 0.001; Table 1) and were younger (*p* = 0.009) than the TD group.

**Table 1.** Sample characteristics

	TD (*N* = 65)			ASD (*N* = 66)			
Sex (male : female)	54 : 11			60 : 6			χ^2^(1) = 1.78, *p* = 0.182
Ethnicity (White : other)	55 : 10			39 : 27			χ^2^(1) = 10.53, *p* = 0.001
	Mean	s.d.	Range	Mean	s.d.	Range	*t* (df)*
Age (months)	42.3	9.00	27–56	38.1	9.34	24–59	*t*(130) = 2.64, *p* = 0.009
MSEL – Visual reception	59.1	11.23	34–80	38.1	16.3	20–80	*t*(115.6) = 8.63, *p* < 0.001
ADOS – CSS	1.6^a^	0.74	1–3	7.1	1.66	4–10	*t*(90.5) = −24.24, *p* < 0.001
PLS – Total	121.8^b^	12.36	94–150	77.0^c^	24.53	50–138	*t*(89.0) = 12.70, *p* < 0.001
VABS – Communication	105.2	8.57	89–127	81.2	14.67	48–112	*t*(105.1) = 11.45, *p* < 0.001
VABS – Socialisation	96.5	7.53	80–112	75.1	10.77	50–106	*t*(116.4) = 13.20, *p* < 0.001
Heart rate	102.90	10.65	80–134	106.06	11.14	78–133	*–*
Heart rate variability	5.88	1.17	3.4–8.9	5.73	1.30	2.9–9.2	*–*

MSEL: Mullen Scales of Early Learning; ADOS – CSS: Autism Diagnostic Observation Schedule – Calibrated Severity Score; PLS: Preschool Language Scale; VABS: Vineland Adaptive Behaviour Scale.

a *N* = 62, ^b^*N* = 60, ^c^*N* = 61.

* Satterthwaite's degrees of freedom is used when groups had unequal variances.

### Protocol

Children were invited to the lab for a diagnostic/cognitive visit (session 1), and then for two additional visits (sessions 2 and 3) for collection of the HR measures and other experimental paradigms. Standardised measures included: (1) the ADOS calibrated severity score (ADOS-CSS) as a measure of autism symptoms; (2) the visual reception domain of the Mullen Scales of Early Learning (MSEL-VR) as an index of non-verbal developmental level; (3) the Preschool Language Scale, Fourth Edition, Total Language (PLS) as a measure of auditory comprehension and expressive verbal communication; (4) and the VABS to assess adaptive communication and socialisation functioning.

### Physiology protocol

During both sessions 2 and 3, children completed a 20-minute computer delivered attention battery at the onset of the session that included nine blocks of different stimuli (see SM 1.2, online Supplementary Fig. S1). Two blocks, the fourth and the seventh block, are of interest for this article and discussed in more detail.

#### Stimuli

Children watched two blocks (fixed order), each with two conditions (random order); the same order of conditions was used for a child at both sessions. Block 1 consisted of the conditions B1-Toy, with videos of activated toys making sounds, and B1-Man, with men singing nursery rhymes, to examine non-social and social responses. Block 2 consisted of the conditions B2-English and B2-Hungarian, with videos of women singing nursery rhymes in English and Hungarian, respectively, to examine social responses to known and unknown languages. Each condition consisted of five individual videos for different toys/songs presented using E-prime 2.0 (see SM 1.2). The next video within each condition started automatically. All data were analysed on the condition level and individual videos were not considered. The conditions had an average length of ~65 s but varied per participant due to discontinuation (e.g. child was upset), buffering time and pauses between videos. All included participants had at least 55 s of data for analysis per condition and participants with elongated conditions who had data that was greater than 10 s over the median length of that condition were excluded (see SM 1.3). Only the first 55 s of each condition were analysed.

#### Autonomic measures

HR data were collected using Biolab (Version 3.0.4, MindWare Technologies LTD; sample rate: 500 Hz, low-pass filter: 0.5 Hz, high-pass filter: 40 Hz) with a lead-II position on chest or back. Digital task markers were sent via E-Prime. Peak identification of the electrocardiogram was done offline using the automatic peak detection in Mindware HRV 3.0.12 and visual inspection (by TB). Data were exported to MATLAB R2014a to segment the data using the digital markers and to make corrections for missing peaks. Missing peaks were corrected by dividing the interbeat interval (e.g. interval was divided by 2 if one peak was missing), up to a maximum of three consecutive missing peaks; conditions with >10% missing peaks or >3 consecutive missing peaks were excluded. The first 55 s of each condition was then analysed in Kubios to extract average HR and HRV data (version 2.2; interpolation rate: 4 Hz, fast Fourier transformation window width: 128 samples, window overlap: 50%). The HRV frequency band was set to 0.24−1.04 to fit the age-appropriate respiration rate (Porges, Doussard-Roosevelt, Portales, & Greenspan, 1996; Skowron, Cipriano-Essel, Gatzke-Kopp, Teti, & Ammerman, 2014).

Children included in the final sample had valid HR data for at least two out of four conditions at session 2 and/or 3 (SM1.3, online Supplementary Table S1). Out of the total eight conditions (four per session), the TD group had data available for 6.8 (s.d. = 1.77) conditions and the ASD group 6.3 (s.d. = 1.90; *t*(129) = 1.78, *p* = 0.077). All mixed models were run with number of available conditions as control variable, but this effect was not significant (all *p*s ⩾0.726) and this variable was excluded from further analysis.

### Statistical analysis

Analyses were run in Stata 16.0 (StataCorp, 2019). First, linear mixed models (*mixed* command with restricted maximum likelihood estimation) compared TD and ASD groups. Fixed effect variables were age, order of conditions, MSEL-VR, condition, group, and the condition-by-group interaction effect. Participant and Session were entered as crossed random effects. The main effect of condition, group and their interaction effect were tested using the *contrast* command and, if significant, compared with *pwcompare* using Bonferroni correction. Normality of residuals was checked using *kdensity*, *pnorm* and *qnorm* plots.

Second, to explore physiological differences between data-driven phenotypic subgroups, we used latent profile analysis (LPA; Spurk, Hirschi, Wang, Valero, & Kauffeld, 2020) in R4.0.3 using LPAtidy (Rosenberg, Beymer, Anderson, Van Lissa, & Schmidt, 2019) to create more homogeneous classes of children within the ASD group, based on MSEL-VR, PLS-Total, VABS communication and socialisation subscales and the ADOS-CSS. LPAtidy uses the *mclust* package, which uses the EM algorithm for maximum likelihood estimation and initialisation is done based on agglomerative hierarchical clustering. Before the LPA, missing data (5 PLS) were imputed using single imputation with *missForest*, based on the available phenotypic variables used in the LPA. We compared 1−5 class solutions (considering sample size); variance was set to equal and covariance to zero. We used the Bayesian Information Criterion (BIC, i.e. lower value), class size (>10%) and interpretation of the created classes to identify the optimal number of classes. A scree plot for the BIC values was used to determine the point at which BIC values started decreasing less. The chosen classes of ASD children and the TD group were again compared using mixed models as above, using the non-imputed dataset.

Third, to explore phenotypic differences between data-driven physiological classes based on the total sample, we again used LPA to identify profiles of variation in HR and HRV across the four conditions. Session 2 and Session 3 data for a participant was entered separately into the same LPA (*N_total_* = 233: *N_ASD−S2_* = 61; *N_ASD−S3_* = 55; *N_TD−S2_* = 61; *N_TD−S3_* = 56). Thus, children could fall into different subgroups for each session. Eight percent (see online Supplementary Table S1 for breakdown of available data per condition and session) of datapoints was missing and were imputed before the LPA using single imputation with *missForest*, based on the available physiological data per session. We considered 1−10 possible classes as it included both the TD and ASD groups and both sessions. As above, we used BIC, class size and interpretation for class selection. We subsequently compared the classes on cognitive skills, social skills and symptom severity, using the non-imputed dataset.

This approach resulted in four classes of children with overall high to low arousal. The classes did not differ on any phenotypic measures but did differ in age (see SM2.4 for the results). Because we were interested in the physiological responses of children to the different conditions, rather than their differences in overall HR and HRV which is influenced by age, we created mean-centred variables based on the imputed dataset. Mean-centred data were created by subtracting a participant's average HR from their HR per condition for each session. The same was done for HRV. Because the data were mean-centred, the mean HR and HRV for each child per session was 0. Negative values represent decreased and positive values represent increased HR/HRV during a condition compared to the child's average HR/HRV. Thus, mean-centred scores reflected whether an individual's physiological arousal during a specific condition was higher or lower compared to the other conditions. Because children could be allocated to different classes at each session, we focussed on the children that fell in the same class at both sessions (i.e. stable). ANOVA and chi-square test were used to compare classes on the phenotypic variables and ratio of children from the ASD/TD, respectively.

## Results

### ASD *v.* TD comparison

#### Heart rate

**Fig. 1.**
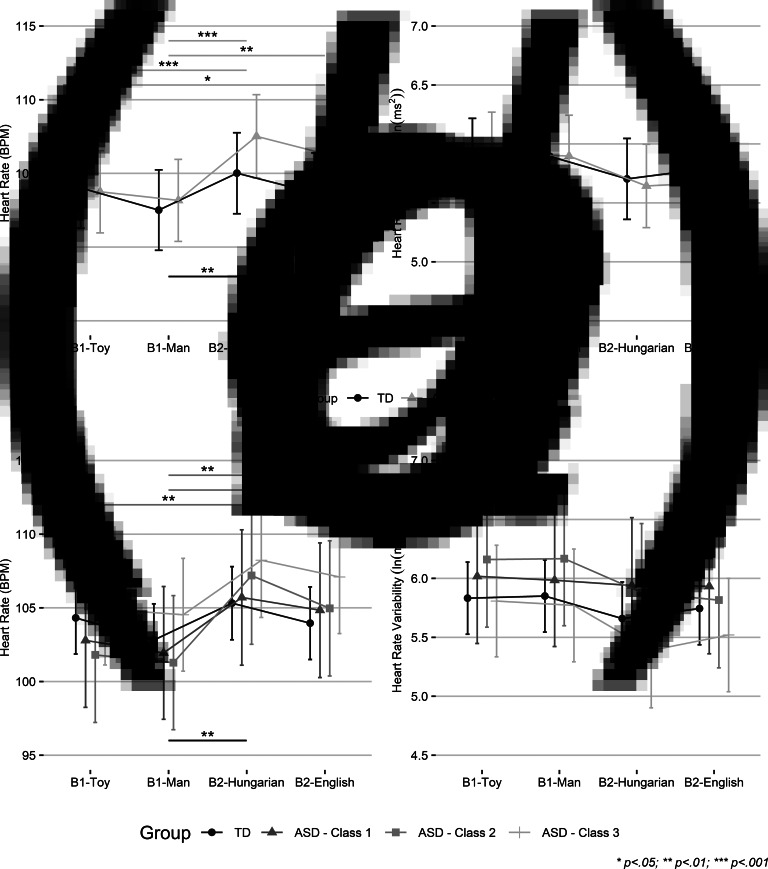
Heart rate and heart rate variability for each condition. All figures are based on the marginal means. Error bars represent 95% confidence interval. (a) Average heart rate for TD *v.* ASD, (b) Average heart rate variability for TD *v.* ASD, (c) Average heart rate for TD *v.* three ASD classes, (d) Average heart rate variability for TD *v.* three ASD classes. Significant differences between conditions for significant interactions are given for (a) TD (below) and ASD (above), (c) TD (below) and ASD-C2 (above).

Model statistics are presented in Table 2 (TD *v.* ASD). Age was a significant predictor of HR, such that older children had lower HR when watching the videos. There was a significant effect of condition (see SM2.1) and a significant group-by-condition interaction effect (Fig. 1a); whilst the TD group showed an increase in HR from B1-Man to B2-Hungarian only [contrast = 2.50, *p* = 0.008, 95% CI (0.45–4.55)], the ASD group showed an increase in HR from B1-Man to the B2-Hungarian and B2-English conditions [respectively, contrast = 4.35, *p* < 0.001, 95% CI (2.20–6.50); contrast = 2.99, *p* = 0.001, 95% CI (0.90–5.08)] and an increase in HR from B1-Toy to the B2-Hungarian and B2-English conditions [respectively, contrast = 3.76, *p* < 0.001, 95% CI (1.62–5.91); contrast = 2.41, *p* = 0.014, 95% CI (0.32–4.50)].

**Table 2.** Model statistics of mixed models for heart rate and heart rate variability for TD *v.* ASD and TD *v.* three ASD classes

	TD *v.* ASD				TD *v.* three ASD classes			
	Heart rate		Heart rate variability		Heart rate		Heart rate variability	
Fixed effects	*B* (s.e.)	*p*	*B* (s.e.)	*p*	*B* (s.e.)	*p*	*B* (s.e.)	*p*
Age in months	**−0.63 (0.09)** CI [−0.80 to −0.45]	**<0.001**	**0.05 (0.01)** CI [0.03–0.08]	**<0.001**	**−0.62 (0.09)** CI [−0.80 to −0.44]	**<0.001**	**0.05 (0.01)** CI [0.03–0.07]	**<0.001**
Order^a^	1.06 (1.55) CI [−1.97 to 4.09]	0.493	0.04 (0.19) CI [−0.33 to 0.42]	0.816	0.99 (1.55) CI [−2.05 to 4.03]	0.524	0.06 (0.19) CI [−0.32 to 0.44]	0.768
MSEL-VR	0.03 (0.06) CI [−0.08 to 0.15]	0.588	−0.005 (0.01) CI [−0.02 to 01]	0.528	–	–	–	–
	χ^2^ (df)	*p*	χ^2^ (df)	*p*	χ^2^ (df)	*p*	χ^2^ (df)	*p*
Condition^b^	**40.40 (3)**	**<0.001**	**14.14 (3)**	**0.003**	**44.37 (3)**	**<0.001**	**12.70 (3)**	**0.005**
Group^c^	0.42 (1)	0.519	0.01 (1)	0.906	1.20 (3)	0.754	1.80 (3)	0.616
Condition × Group	**9.26 (3)**	**0.026**	1.30 (3)	0.729	11.27 (9)	0.258	4.53 (9)	0.874
Random effects	Estimate	s.e.	Estimate	s.e.	Estimate	s.e.	Estimate	s.e.
Session	0.02	0.16	1.04 × 10^−17^	–	0.02	0.16	1.35 × 10^−17^	–
ID	79.93	10.76	1.21	0.17	79.99	10.80	1.21	0.17
Residual	32.55	1.72	0.58	0.03	32.74	1.73	0.58	0.03

Significant effects are in bold, CI: 95% confidence interval, MSEL-VR: Mullen Scales of Early Learning – Visual reception.

a Order of conditions: Toy-Man/Hungarian-English and Man-Toy/English-Hungarian.

b Condition: B1-Toy, B1-Man, B2-Hungarian and B2-English.

c Group: TD and ASD (left columns) or TD, ASD-C1, ASD-C2 and ASD-C3 (right columns).

#### Heart rate variability

Age was a significant predictor of HRV; older children had higher HRV when watching the videos (Table 2). There was a significant effect of condition; HRV was higher during B1-Toy compared to B2-Hungarian [contrast = −0.23, *p* = 0.016, 95% CI (−0.42 to −0.03)] and B1-Man compared to B2-Hungarian [contrast = −0.22, *p* = 0.018, 95% CI (−0.42 to −0.02)]. No other effects were significant.

#### Summary

Both HR and HRV were modulated by the video conditions. The ASD group showed an increase in HR from Block 1 Toy or Man conditions to the Block 2 videos (English and Hungarian); this increase was less pronounced in the TD group.

### Phenotypic classes

A three-class solution was chosen based on the lowest BIC value (BIC = 2368.07; entropy = 0.91; see SM 2.2, online Supplementary Table S2). The five-class solution was considered as it had a lower sample-size adjusted BIC value, however, the three-class solution led to more distinct profiles, facilitating interpretation. Cognitive profiles are presented in Table 3 (for boxplots see SM 2.3, online Supplementary Fig. S2). The first class (ASD-C1, *N* = 19) had cognitive, adaptive and language scores closest to the TD group, but relatively high symptom severity on the ADOS. The last class (ASD-C3, *N* = 28) reflected a ‘most impaired’ subgroup with lowest scores on non-verbal ability, language and socialisation, most ASD symptoms, and younger age than TD. The second class (ASD-C2, *N* = 19) had cognitive, language and adaptive scores that were intermediate in level, but had the lowest symptom severity score of the three ASD classes.

**Table 3.** ANOVA comparing phenotypic ASD classes and TD group

	TD *N* = 65	ASD (C1) *N* = 19	ASD (C2) *N* = 19	ASD (C3) *N* = 28	*F* (*p*)
Age in months	42.31^a^	42.00^a, b^	37.42^a, b^	35.86^b^	4.17 (0.008)
MSEL – VR	59.12^a^	57.32^a^	40.95^b^	23.07^c^	100.75 (<0.001)
ADOS – CSS	1.58^a^	7.00^b^	5.89^c^	7.89^b^	245.06 (<0.001)
PLS – Total	121.77^a^	112.25^b^	75.44^c^	57.22^d^	242.81 (<0.001)
VABS – Com	105.22^a^	98.42^b^	78.47^c^	71.43^c^	112.87 (<0.001)
VABS – Soc	96.52^a^	82.74^b^	74.00^c^	70.71^c^	74.28 (<0.001)

*Note.* Shading from light to dark: typical to most atypical score.

Groups marked with different superscript letters (a, b, c, d) differed significantly with Bonferroni correction applied (*p* < 0.05).

MSEL -VR: Mullen Scales of Early Learning – Visual reception; ADOS – CSS: Autism Diagnostic Observation Schedule – Calibrated Severity Score; PLS: Preschool Language Scale; VABS: Vineland Adaptive Behaviour Scale, Com: Communication, Soc: Socialisation.

#### HR in phenotypic classes

The same mixed model was run with the TD group and the three ASD classes (Table 2, Fig. 1c) omitting MSEL-VR (used in class creation). There was no overall condition by group effect but because this was present in the first model of HR, we repeated the analysis including the TD group and one of the ASD subgroups at a time (see SM 2.2, online Supplementary Table S3). Only ASD-C2 showed a significant effect, specifically an interaction between group (TD *v.* ASD-C2) and condition (χ^2^(3) = 10.47, *p* = 0.015). Only the ASD-C2 showed an increase in HR from B1-Toy to B2-Hungarian [contrast = 5.37, *p* = 0.001, 95% CI (1.65–9.09)] and from B1-Man to B2-English [contrast = 3.67, *p* = 0.037, 95% CI (0.14–7.20)]. Both TD and ASD-C2 showed an increase in HR from B1-Man to B2-Hungarian [contrast = 2.50, *p* = 0.008, 95% CI (45–4.56), contrast = 5.91, *p* = 0.001, 95% CI (1.85–9.97), respectively]. Comparing ASD-C2 to the other two ASD classes did not show a group or interaction effect (see SM 2.3, online Supplementary Table S4).

#### HRV in phenotypic classes

As with the first HRV model, only age and condition were significant predictors of HRV (Table 2; Figure 1d).

#### Summary

Three classes of children with ASD were identified based on their phenotypic profile. Only the ASD-C2 class, which had phenotypic scores between the other two ASD classes, showed a HR response that was significantly different from the TD group, such that an increase in HR was observed from B1-Toy to B2-Hungarian and from B1-Man to B2-English.

### Physiological classes

**Fig. 2.**
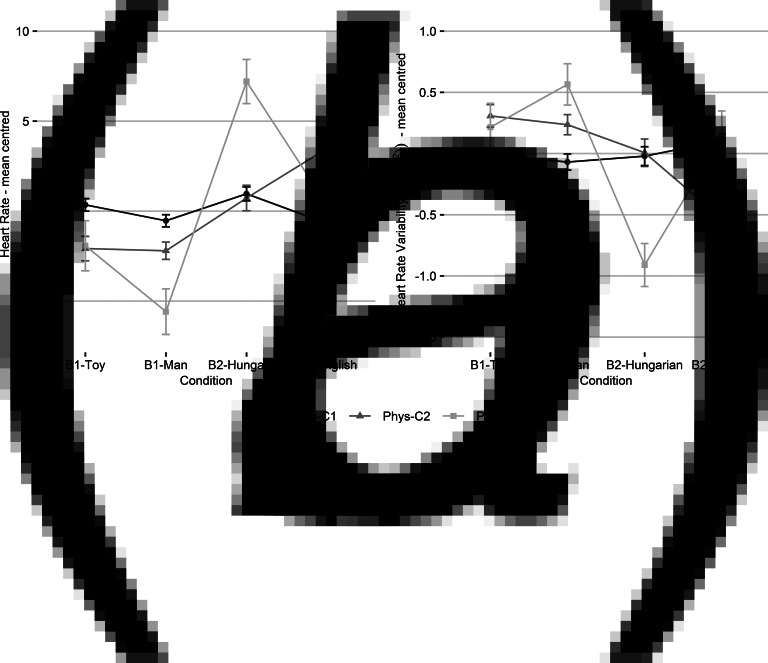
Latent profile classes for three-class solution based on the mean-centred physiological data of the total sample for (a) heart rate and (b) heart rate variability. Error bars represent 95% confidence interval.

The six-class model had the lowest BIC but individual class size was small (smallest class 3%). Because the difference in BIC values started to decrease after three classes and this model had larger class sizes (11%), this model was chosen for the mean-centred HR and HRV variables (BIC = 5447.03, Entropy = 0.80; SM2.2, online Supplementary Table S2). Phys-C1 (*N* = 145) showed only small changes in HR and HRV between the four conditions. In contrast, Phys-C2 (*N* = 63) showed higher HR and lower HRV during Block 2 compared to Block 1 and Phys-C3 (*N* = 25) showed a response that differed by condition (arousal decrease to the B1-Man and increase to the B2-Hungarian videos, Fig. 2).

#### Stable physiological classes

As children could be allocated to different classes at each session, we focus on the 61% of children who fell in the same physiological class at both sessions (Phys-C1_stable_: *N* = 43, Phys-C2_stable_: *N* = 17, Phys-C3_stable_: *N* = 2; SM 2.5, online Supplementary Table S6). As only two children were in Phys-C3_stable_, we did not consider this class any further. The Phys-C2_stable_ group has lower scores on the MSEL-VR, PLS and VABS communication (SM 2.5, online Supplementary Table S7) and had more children from the ASD group (76%) than Phys-C1_stable_ (44%, χ^2^(1) = 5.10, *p* = 0.024). Group comparisons per session can be found in SM 2.5.

## Discussion

We investigated the physiological responses of preschool-aged children with and without ASD to non-social and social videos. We measured HR and high-frequency HRV responses, compared diagnostic groups and used LPA to identify more homogeneous groups of children based on their phenotypic and physiological profiles. No differences in overall HR and HRV were observed between preschoolers with and without ASD. However, the diagnostic groups did differ in their response to social videos presented later in the battery, with the ASD group showing a larger increase in HR to a comprehensible social video presented later in the session than the TD group. Looking at the latent profiles, this was specifically the case for the class of children who showed difficulties with non-verbal, communication and socialisation skills, but who showed relatively lower levels of ASD symptomatology (ASD-C2). Similarly, children who fell reliably in a class showing higher HR during the second, compared to the first block (Phys-C2) had lower non-verbal and verbal skills. Taken together, these results suggest there may be a subgroup of children with ASD who have poorer cognitive abilities and more fragile attention engagement to social stimulation.

There was no overall difference in HR or HRV between the ASD and TD groups, in line with Bazelmans et al. (2019). Both groups showed increased arousal to the Hungarian video. This is in line with the findings by Pempek et al. (2010). Although Pempek et al. (2010) did not find differences in HR between comprehensible *v.* non-comprehensible videos, they did find that from 18 months, children had shorter look durations to a non-comprehensible video. In turn, shorter looks were associated with higher HR. Our physiology data suggest that children in our sample showed higher levels of arousal during a video they could not comprehend, which could be an indication of reduced engagement with the video.

Group difference were observed in the modulation of HR by condition such that the TD group showed an increase in HR from the Man to the Hungarian video only, whereas the ASD group showed an increase in HR from both the Toy and Man (Block 1) videos to the Hungarian and English (Block 2) videos. This larger HR reaction in the ASD group could suggest that although all children are less engaged with the incomprehensible Hungarian video, the ASD group shows less re-engagement with the English video despite showing no differences to the TD group in the earlier-presented Man video. Following this interpretation, this pattern would represent a failure to re-engage attention to social stimuli after it has waned across the session in the ASD group. In an exploratory post-hoc analysis including ‘calm’ animal/nature videos presented before and after the Hungarian/English videos (reported in Bazelmans et al., 2019; SM3.1, online Supplementary Fig. S4), we observed a general trend for HR to increase over time; however, only the TD group showed a decrease in HR from the calm video preceding the second block (calm 3) to the English video. This finding is again consistent with more difficulty engaging with comprehensible social content in the ASD group relative to the TD group, particularly when attention is waning.

Next, we created more homogenous groups of children with ASD using LPA. The ASD-C1 group had cognitive and social skills that were closest to those of the TD children, but relatively higher scores on symptom severity, whereas ASD-C2 had lower cognitive and social skills, but also slightly lower symptom severity scores. Given our preschool age range, children in ASD-C1 with relatively good cognitive skills perhaps need to have more severe ASD symptoms in order to be diagnosed early. Interestingly, we did not find that the most clinically impaired group (ASD-C3) had the highest arousal levels. Rather, ASD differences (decreased re-engagement to the later English video) seemed most clearly driven by the ASD-C2 group.

These results were broadly confirmed by the LPA based on physiological data. Only two classes with sufficient participant numbers and stability across test−retest sessions were identified: no differences between conditions, and an increase between the Block 1 (Toy/Man) and Block 2 (Hungarian/English) videos. These subgroups differed in cognitive skills such that children who showed higher HR to Block 2 videos had lower non-verbal and verbal skills than children who did not show this increase in HR, but they did not differ on social skills or ASD symptoms.

Taking the data from the three analyses together, children with ASD with moderate cognitive delays in our sample show increased HR during videos with comprehensible social content later in the session. Although the two groups seem to initially respond similarly on a physiological level to the social content, as no differences were observed between the toy and man videos, the increase in HR suggests the ASD group has difficulties regulating their physiological response to social information over time. These results may suggest difficulties in their ability to re-engage when attention is diminishing. This increase in arousal, whether this reflects an increase in stress or a decrease in attention, may have an impact on their acquisition of nonverbal/verbal skills. Vice versa, their poorer understanding of the language in the social videos might make them less inclined to reorient to this information. Either way, this subgroup of children with ASD, who are potentially more fragile in terms of their attention engagement, may be missing out on key learning opportunities. The change in their arousal from the first to the second part of the paradigm, could reflect a difficulty to maintain engagement or re-engage with social information over longer periods of time and should be considered in educational and therapeutic contexts to optimise their efficacy.

### Limitations

In the current study, we use physiological arousal as a measure of attentional engagement, however we do not include looking behaviour. Additional information about looking patterns or assessment of post-exposure familiarity could inform us of the extent to which attentive, memory, or linguistic processing are related to these findings. Nevertheless, arousal has been found to predict looking behaviour during dynamic videos (de Barbaro et al., 2017) and our videos present both visual and auditory information and visual disengagement may not necessarily mean the child is not processing or engaged with the auditory information.

Measuring social engagement and processing in ASD is challenging and it is difficult to distinguish what is an atypical but adaptive response to their environment. It should also be noted that children with ASD may be differently engaged with screen-based stimuli than they would be during direct contact with other people. For example, children with ASD are more exposed to screens (Slobodin, Heffler, & Davidovitch, 2019) and may use this as a tool to learn and imitate social behaviours (Shane & Albert, 2008). It has also been shown that reduced attention to the face is mainly observed during screen-based viewing compared to live interaction and that in individuals with ASD visual attention to the screen is not associated with attention during live interactions (Grossman, Zane, Mertens, & Mitchell, 2019). To generalise these results to face-to-face situations, this study should be repeated using live presentation and interaction. It is equally important to understand responses to a range of different people, such as familiar adults, peers, and others with autism, to get from a better understanding of social engagement (Davis & Crompton, 2021).

We included a group of young children with an ASD diagnosis from a narrow age range (2–4 years) with a wide range of non-verbal, verbal and social competencies, and successfully created classes of children based on their abilities and physiology. Findings may, however, be only representative of boys with ASD as only 9% of our ASD sample consisted of girls, which is less than we would expect in the general population (Loomes, Hull, & Mandy, 2017).

We used LPA to understand whether classes exist, though it should be noted that our sample is relatively small for these types of analyses. Moreover, the decision on the optimal number of classes for each LPA was not always clear cut. For example, for the mean-centred physiology LPA, the two-class model could have been chosen based on a higher entropy. This would result in two classes similar to Phys-C1 and Phys-C3, without Phys-C2. Nevertheless, the three-class model led to three distinct profiles, that matched the interpretation of the phenotypic clusters. Furthermore, the findings from the phenotypic LPA can provide a starting point for other studies to use cognitive and symptom characteristics to find more homogeneous groups of children with ASD.

## Conclusions

The present study confirms previous observations of minimal overall atypicality in HR or HRV in preschoolers with ASD. However, subgrouping analyses indicated that a subset of the ASD group (29% of the total) show decreased re-engagement to a comprehensible social video presented as attention wanes. This physiological modulation profile appears most associated with nonverbal and verbal skills, rather than social symptoms or abilities. Limited cognitive abilities in a subgroup of young children with ASD may be associated with poor attention to social stimuli when the child's attention is more fragile (e.g. later in a session). Future investigations of the neurocognitive mechanisms underpinning ASD may require nuanced approaches that include subgrouping, and consideration of the interaction between child engagement state and experimental manipulation. The identification of a subgroup of children with ASD who have particularly fragile attention skills could be informative for considering child state in intervention and assessment programmes.

## Supplementary material

For supplementary material accompanying this paper visit https://doi.org/10.1017/S0033291721003172. click here to view supplementary material

## Authors’ information

Sheila Ghods and Emily J. H. Jones were at the Seattle Children's Research Institute, Seattle, WA, USA at the time of the study. Sheila Ghods is currently at the University of California San Francisco, San Francisco, CA, USA. Emily J. H. Jones is at Birkbeck College, University of London, London, UK.
