# Supplementary material for: Identifying phenotypic and physiological subgroups of preschoolers with autism spectrum disorder
Source: Psychol Med. 2021 Sep 17;53(4):1592–602. doi: 10.1017/S0033291721003172 (PMC10009381; doi:10.1017/S0033291721003172)
Supplement: Supplementary file 1 [file S0033291721003172sup001.docx]

# Supplementary Materials

1. **Methods**
   1. **Included versus excluded ASD participants**

There were no significant differences between the 19 children excluded in the ASD group based on missing physiological data compared to the 66 included children: ADOS (*t*(83) = 0.34, *p* = .735), Visual Reception (*t*(83) = -0.44, *p* = .661), Preschool Language Scale (*t*(75) = 0.35, *p* = .725), Vineland Communication (*t*(83) = 0.02, *p* = .983) and Vineland Socialisation (*t*(83) = -0.33, *p* = .745).

- 1. **Order & Stimuli**

The total screen-based battery consisted of 9 blocks alternating calm viewing and active attention. Figure S1 shows the order of all videos in the screen-based battery. Block 1 and Block 2 in Figure S1 are part of the main analysis of this article. The blocks labelled ‘other’ are not part of this article and therefore are not further discussed. The blocks labelled ‘calm’ are presented in Bazelmans et al. (2019) and are used in an exploratory analysis in the Discussion section (see also SM 4.1). The calm videos consisted of animal videos accompanied by classical music.


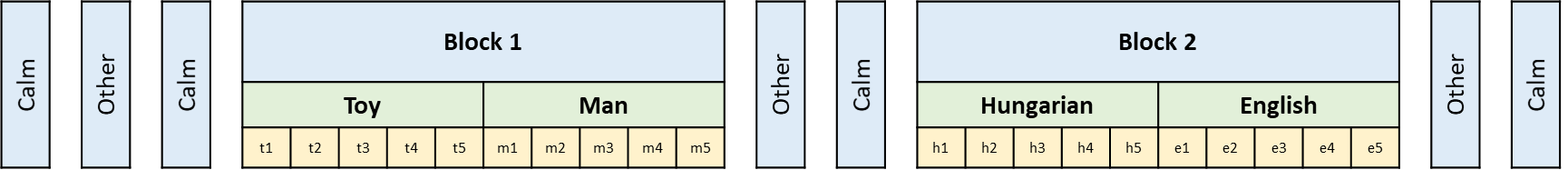


Figure S1. Order of blocks in screen-based battery. Blue: blocks, green: condition, yellow: video.

The order of Blocks 1 and 2 was consistent, but the order of the conditions within each block was counterbalanced between participants and kept constant for each participant across the two session. Thus, half of the participant had the order Toy – Man – Hungarian – English, the other half had the order Man – Toy – English – Hungarian. Note: One child received the order Toy-Man-Hungarian-English at the first physiological session (Session 2) and the order Man-Toy-English-Hungarian at the second physiological session (Session 3).

Each condition consisted of five individual videos which were 5 to 20 seconds in length. There were two different Batteries (A and B). Half the children saw Battery A at the first phycological session (Session 2) and Battery B at the second physiological session (Session 3). The other half saw Battery B at Session 2 and Battery A at Session 3. The condition **Toy** consisted of 2 mechanical child toys that included lights and sounds. These were presented in alternating order (Battery A: Toy1 – Toy2 – Toy1 – Toy2 – Toy1, Battery B: Toy2- Toy1 – Toy2 – Toy1 – Toy2) and the melodies the toys played were different for Battery A and B. The condition **Man** consisted of 2 adult males (M1 and M2) singing nursery rhymes. The nursery rhymes where always presented in the order: ‘The farmer in the dell’, ‘Hickory dickory dock’, Twinkle twinkle little star’, ‘Mary had a little lamb’, ‘Baa baa black sheep’ but the order of the two men varied per session (Battery A: M1 – M2 – M1 – M2 – M1 or Battery B: M2 – M1 – M2 – M1 – M2).

The conditions Hungarian and English consisted of 2 adult females (F1 and F2) who were native / fluent speakers of both English and Hungarian. Hungarian was used as it is structurally and historically dissimilar from English and no child in the study had pre-exposure to Hungarian. The **English** nursery rhymes in Battery A were: ‘Hot cross buns’, It’s raining, it’s pouring’, ‘If you’re happy and you know it’, ‘Are you sleeping Brother John?’, ‘Old MacDonald had a farm’. Battery B had the same nursery rhymes in reversed order. The same was done for the **Hungarian** nursery rhymes. The order of the females was in Battery A: F1 – F2 – F1 – F2 – F1 for English and F2 – F1 – F2 – F1 – F2 for Hungarian. The opposite order was used for Battery B (starting with F2 for English and F1 for Hungarian). Note, in one of the Batteries, markers were not sent by E-Prime due to a programming error. This affected all participants at one of the two sessions. The start times of the missing markers were calculated by coding the relative onset times post-acquisition in Mangold.

All videos are available upon request.

- 1. **Data available per condition**

While the next video within each condition started automatically after buffering, the experimenter was able to pause between videos if the child was not attending. The experimenter could end the condition early if the child was too distressed or distracted. Because of the varied duration between individual videos, due to buffering or pauses, the total length for each condition varied between participants. All conditions were therefore capped at 55 seconds for analysis. To allow for some variation but take out participants that had long breaks between the videos within a condition, data was removed for conditions with a total length over 10 seconds of the median (21 conditions: ASD: Toy = 2, Hungarian = 10; English = 1; TD: Toy = 1, Man = 1, Hungarian = 5; English = 1)). Conditions which were 54 seconds or shorter, i.e. due to early termination of the task, where also excluded (7 conditions: ASD: Man = 2, Hungarian = 2; TD: Toy = 1, Hungarian = 2).

Of the 131 children included in the study, 50 of 66 children in the ASD group and 52 of 65 children in the TD group had HR data available at both session 2 and 3, which were 2 to 6 weeks apart (Median: 17 days, range 10-40 days).

Table S1. *N of children with available physiological data per condition.*

|  | **ASD (*N* = 66)** | |  | **TD (*N* = 65)** | | **Condition length** |
| --- | --- | --- | --- | --- | --- | --- |
|  | *Session 2* | *Session 3* |  | *Session 2* | *Session 3* | *Median (in seconds)* |
| **N per session** | **61** | **55** |  | **61** | **56** |  |
| Toy | 57 | 51 |  | 59 | 55 | 65.5 |
| Man | 57 | 51 |  | 61 | 53 | 63.2 |
| Hungarian | 49 | 45 |  | 54 | 51 | 68.3 |
| English | 54 | 49 |  | 58 | 53 | 67.1 |

1. **Results**
   1. **ASD versus TD comparison: Main effect of condition**

HR increased from B1-Toy to B2-Hungarian (contrast =2.38, *p* <.001, 95% CI [.90, 3.87]), B1-Man to B2-Hungarian (contrast = 3.42, *p* <.001, 95% CI [1.94, 4.91]) and B1-Man to B2-English (contrast =2.07, *p* =.001, 95% CI [0.61, 3.52]).

- 1. **Model fit statistics for latent profile analyses**

Table S2. *Latent Profile Analyses Model fits for phenotypic and physiological classes*

| # classes | Log-likelihood | AIC | BIC | saBIC | Entropy | Smallest class | Largest class |
| --- | --- | --- | --- | --- | --- | --- | --- |
| Latent profile analysis for phenotypic data (*N* = 66) | | | | | | | |
| 1 | -1228.49 | 2476.97 | 2498.87 | 2467.39 | 1.00 |  |  |
| 2 | -1157.04 | 2346.08 | 2381.12 | 2330.75 | 0.95 | 35% | 65% |
| **3** | **-1137.95** | **2319.90** | **2368.07** | **2298.81** | **0.91** | **29%** | **42%** |
| 4 | -1127.09 | 2310.18 | 2371.49 | 2283.34 | 0.88 | 17% | 39% |
| 5 | -1118.86 | 2305.72 | 2380.16 | 2273.13 | 0.93 | 11% | 41% |
| Latent profile analysis for physiology data (*N* = 233) | | | | | | | |
| 1 | -5279.06 | 10590.12 | 10645.34 | 10594.62 | 1.00 |  |  |
| 2 | -4702.05 | 9454.10 | 9540.38 | 9461.14 | 0.92 | 40% | 60% |
| 3 | -4383.57 | 8835.15 | 8952.48 | 8844.72 | 0.94 | 19% | 52% |
| **4^†^** | **-4210.34** | **8506.68** | **8655.08** | **8518.79** | **0.94** | **14%** | **37%** |
| 5 | -4125.10 | 8354.21 | 8533.66 | 8368.85 | 0.94 | 8% | 36% |
| 6 | -4078.25 | 8278.49 | 8489.01 | 8295.67 | 0.92 | 6% | 25% |
| 7 | -4040.86 | 8221.72 | 8463.30 | 8241.43 | 0.93 | 4% | 23% |
| 8 | -4002.58 | 8163.16 | 8435.80 | 8185.40 | 0.93 | 4% | 24% |
| 9 | -3966.38 | 8108.76 | 8412.45 | 8133.53 | 0.93 | 3% | 21% |
| 10 | -3913.06 | 8020.11 | 8354.86 | 8047.42 | 0.93 | 1% | 16% |
| Latent profile analysis for centered physiology data (*N* = 233) | | | | | | | |
| 1 | -2793.85 | 5619.71 | 5674.92 | 5624.21 | 1.00 |  |  |
| 2 | -2690.65 | 5431.31 | 5517.58 | 5438.35 | 0.92 | 12% | 88% |
| **3^†^** | **-2630.85** | **5329.70** | **5447.03** | **5339.27** | **0.80** | **11%** | **62%** |
| 4 | -2590.15 | 5266.29 | 5414.69 | 5278.40 | 0.82 | 3% | 57% |
| 5 | -2556.16 | 5216.33 | 5395.78 | 5230.97 | 0.82 | 6% | 56% |
| 6 | -2514.09 | 5150.18 | 5360.70 | 5167.36 | 0.84 | 3% | 50% |
| 7 | -2494.46 | 5128.92 | 5370.49 | 5148.62 | 0.80 | 1% | 38% |
| 8 | -2461.47 | 5080.94 | 5353.57 | 5103.18 | 0.79 | 1% | 34% |
| 9 | -2448.91 | 5073.82 | 5377.51 | 5098.60 | 0.80 | 1% | 34% |
| 10 | -2429.47 | 5052.94 | 5387.69 | 5080.25 | 0.81 | 1% | 33% |
| AIC: Akaike information criterion, BIC: Bayesian information criterion, saBIC: Sample size adjusted Bayesian information criterion.  The chosen model is in bold.  † Although some subsequent models had lower BIC values, the decrease in BIC started to flatten after the chosen model and subgroup size became small. | | | | | | | |

- 1. **Latent profile analysis based on phenotypic data**

*
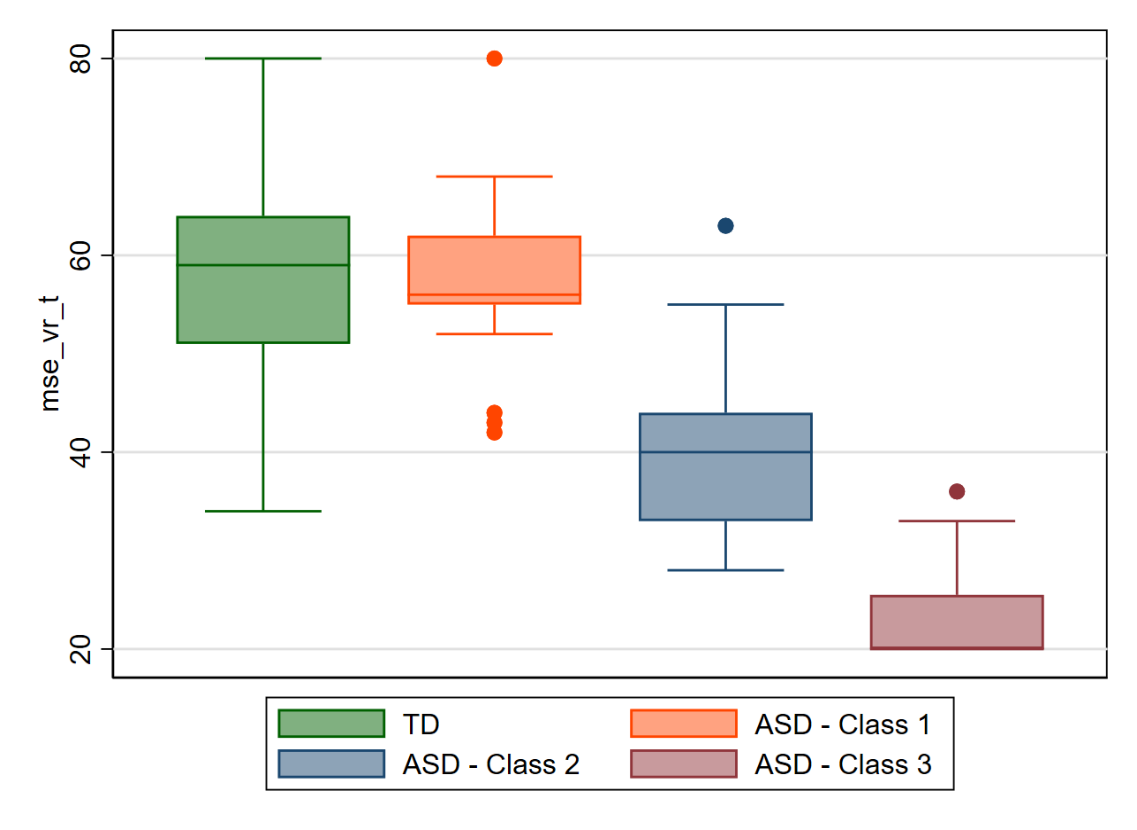
*

Figure S2. Cognitive profiles of the four ASD classes and the TD group.

Table S3. *Mixed models for the typically developing children (TD) and each phenotypic class separately*.

|  | TD vs ASD-C1 | | TD vs ASD-C2 | | TD vs ASD-C3 | |
| --- | --- | --- | --- | --- | --- | --- |
| Fixed Effects | B (SE) | p | B (SE) | p | B (SE) | p |
| Age in months | **-0.57 (0.11)** | **<.001** | **-0.59 (0.11)** | **<.001** | **-0.63 (0.11)** | **<.001** |
| Order of conditions | -0.11 (1.98) | .954 | -0.06 (2.04) | .978 | -1.56 (1.90) | .413 |
|  | χ^2^ (df) | p | χ^2^ (df) | p | χ^2^ (df) | p |
| Condition | **16.48 (3)** | **<.001** | **32.36 (3)** | **<.001** | **18.75 (3)** | **<.001** |
| Group | 0.03 (1) | .869 | 0.00 (1) | .955 | 0.81 (1) | .368 |
| Condition x Group | 2.89 (3) | .409 | **10.47 (3)** | **.015** | 3.94 (3) | .268 |
| Random Effects | Estimate | S.E. | Estimate | S.E. | Estimate | S.E. |
| Session | 1.30e^-15^ | 1.11^e-14^ | 1.11e^-08^ | - | 0.02 | 0.20 |
| ID | 90.01 | 14.60 | 81.95 | 12.70 | 77.84 | 12.45 |
| Residual | 29.73 | 2.16 | 26.73 | 1.71 | 32.81 | 2.03 |

Table S4. *Mixed models for ASD-C2 versus ASD-C1 and C3*.

|  | ASD-C2 vs ASD-C1 | | ASD-C2 vs ASD-C3 | |
| --- | --- | --- | --- | --- |
| Fixed Effects | B (SE) | p | B (SE) | p |
| Age in months | **-0.61 (.16)** | **<.001** | **-0.74 (0.14)** | **<.001** |
| Order of conditions | 5.78 (2.57) | .024 | 3.59 (2.42) | .138 |
|  | χ^2^ (df) | p | χ^2^ (df) | p |
| Condition | **27.31 (3)** | **<.001** | **26.42 (3)** | **<.001** |
| Group | 0.05 (1) | .824 | 0.79 (1) | .375 |
| Condition x Group | 1.41 (3) | .704 | 1.37 (3) | .712 |
| Random Effects | Estimate | S.E. | Estimate | S.E. |
| Session | 5.77e^-19^ | - | 0.20 | .72 |
| ID | 74.46 | 19.70 | 60.97 | 14.71 |
| Residual | 32.83 | 3.34 | 38.70 | 3.56 |

- 1. **Latent profile analysis based on physiological data**

The latent profile analysis (LPA) was first done on the raw, imputed HR and HRV data. To create the physiological classes, data from each session was entered as a separate entry, leading to an N of 233 (see ‘Statistical Analysis’ in the main text). The four-class solution was chosen based on the results of the LPA. Although the BIC (8655.08; Entropy =.94) did decrease in the subsequent models, this decrease became smaller, and the smallest group size became smaller than 10% (SM 2.1, Table S2 for model fit details). The four classes could be interpreted as overall high (Class 1) to low (Class 4) arousal (*N* = 32, 56, 80 and 54 respectively, Figure S3), without any differences in modulation of physiology per video. These classes were associated with age, but not cognitive skills, social skills or symptom severity. Exploring the 6-class model showed a similar pattern of results with classes ranging from high to low arousal.

It should be noted that participants did not fall in the same class at each session (χ^2^(9) = 55.18, *p* = <.001). Forty-seven percent of children fell in the same class at both sessions, 49 percent fell in a class that was one group higher or lower compared to their previous session (Table S5).


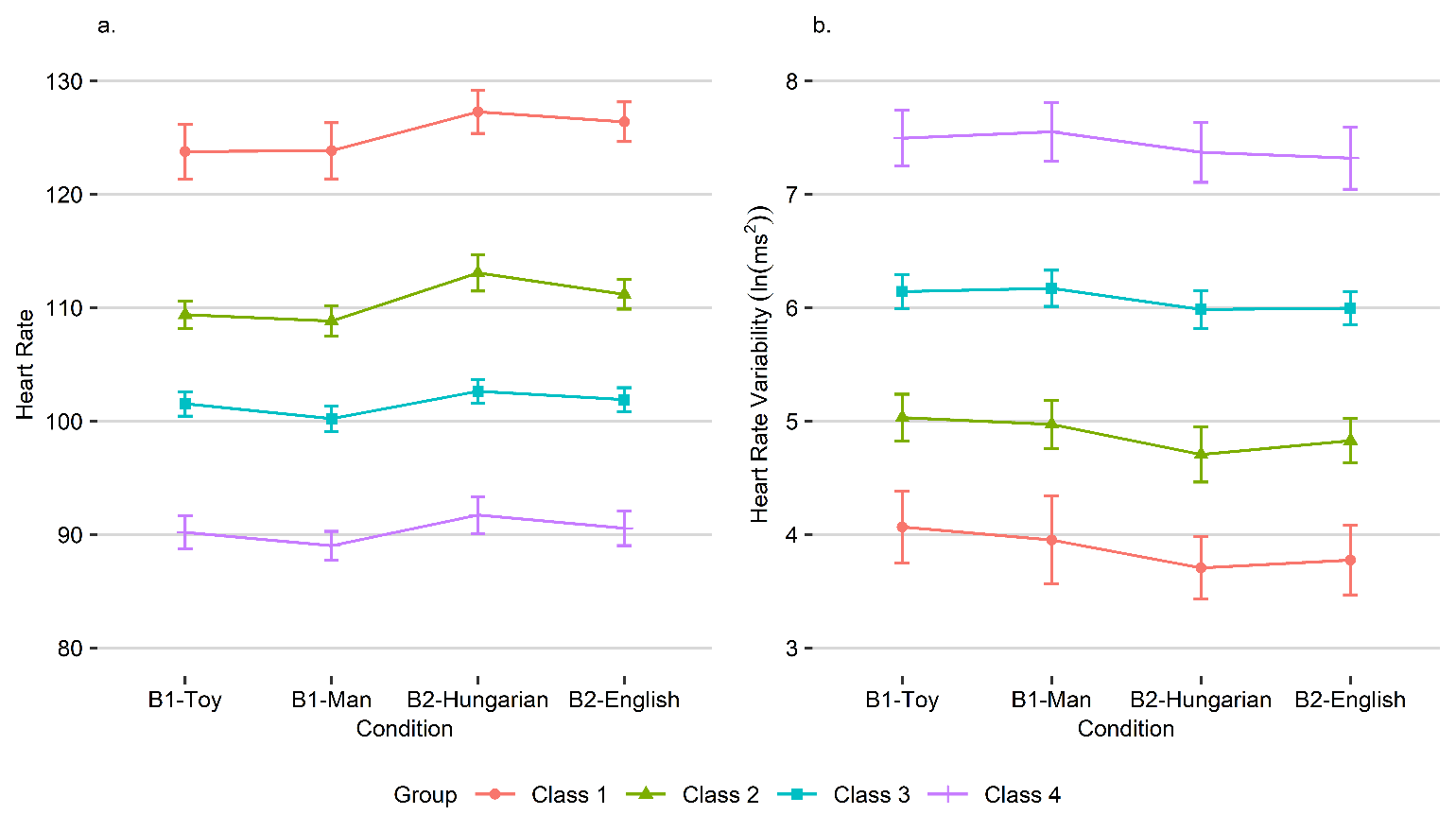


Figure S3. Latent profile classes for four-class solution based on the physiological data for a. heart rate and b. heart rate variability. Error bars represent 95% confidence interval.

Table S5. *Physiological classes at session 2 and 3.*

| Classes Session 2 | Classes Session 3 | | | |
| --- | --- | --- | --- | --- |
|  | 1 | 2 | 3 | 4 |
| 1 | **8** | 3 | 2 | 1 |
| 2 | 5 | **12** | 8 | 1 |
| 3 | 1 | 13 | **15** | 10 |
| 4 | 0 | 0 | 10 | **13** |
| Note: bold are participants that remained in the same class | | | | |

At Session 2, ANOVAs comparing classes did not show group differences on MSEL-VR, PLS-total, VABS communication, VABS socialisation and ASD severity scores (all *p* ≥ .430). Age was significantly different between the groups (*F*(3,118) = 10.59, *p* <.001), such that younger children had higher HR and lower HRV. The two youngest (Classes 1 and 2) and two oldest (Classes 3 and 4) classes did not differ from each other, but all other contrasts were significant (all *p* ≤ .026). There were no differences in the number of children from the ASD and TD group in each of the four physiological classes (χ^2^(3) = 2.37, *p* = .500).

Comparing phenotypic scores of children based on the class they belonged to at Session 3 led to comparable results. No group differences were found for ANOVAs comparing classes on MSEL-VR, language, VABS socialisation and ASD severity scores; VABS communication skills *p* was .051, with class 4 having better communication skills compared to class 1. Age was significantly different between the groups (*F*(3,107) = 12.93, *p* <.001), such that younger children had higher HR and lower HRV. Classes 1 and 2 and Classes 2 and 3 did not differ from each other, but all other contrasts were significant (*p* ≤ .043) Again, there were no difference in number of children from each group in each class (χ^2^(3) = 4.03, *p* = .258). Thus, subgroups can be identified based on physiological data and these reflect classes of children with low to high overall arousal levels, but this is mainly related to the age of the participants.

- 1. **Latent profile analysis based on centred physiological data**

There was a difference between which class a participant fell at each session (χ^2^(4) = 16.12, *p* = .003). Sixty-one percent of children fell in the same class at both sessions (Table S6).

Comparisons between the three classes on phenotypic measures for session 2 and 3 can be found in Table S7. Only at session 3 there was a significant difference in PLS language score (*F*(2,102) =3.28, *p* =.04) such that Phys-C2 had lower language skills compared to Phys-C1 (*p* =.039). None of the other ANOVAs was significant. There was also no difference in the number of children from the ASD and TD group in each class at either session (*p* ≥.174).

Table S6. *Physiological classes using centred data at session 2 and 3.*

| Classes Session 2 | Classes Session 3 | | |
| --- | --- | --- | --- |
|  | 1 | 2 | 3 |
| 1 | **43** | 10 | 6 |
| 2 | 13 | **17** | 1 |
| 3 | 8 | 2 | **2** |
| Note: bold are participants that remained in the same class | | | |

Table S7. *Means for physiological classes and group comparison using ANOVA and t-test.*

|  | **Session 2** | | | | **Session 3** | | | | **Stable between Session**  **2 & 3** | | |
| --- | --- | --- | --- | --- | --- | --- | --- | --- | --- | --- | --- |
|  | **C1**  ***N* = 76** | **C2**  ***N* = 33** | **C3**  ***N* = 13** | **F (*p*)** | **C1**  ***N* = 69** | **C2**  ***N* = 30** | **C3**  ***N* = 12** | **F (*p*)** | **C1^­­^_stable_**  ***N* = 43** | **C2_stable_**  ***N* = 17** | ***t* (*p*)** |
| Age in months | 41.63 | 39.06 | 39.46 | 0.98 (.377) | 40.49 | 39.07 | 36.33 | 1.09 (.340) | 40.88 | 38.29 | 1.03 (.309) |
| MSEL – VR | 49.93 | 44.00 | 51.08 | 1.49 (.230) | 51.41 | 43.37 | 47.42 | 2.38 (.098) | **51.84^a^** | **40.24^b^** | **2.33 (.023)** |
| ADOS – CSS | 4.16 | 4.84 | 4.54 | 0.58 (.564) | 4.14 | 4.43 | 5.50 | 1.07 (.347) | 4.10 | 5.53 | -1.66 (.102) |
| PLS – Total | 103.23 | 89.07 | 98.75 | 2.36 (.099) | **104.77^a^** | **88.00^b^** | **96.25** | **3.28 (.042)** | **105.43^a^** | **76.00^b^** | **3.42 (.001)** |
| VABS – Com | 94.99 | 90.27 | 90.08 | 1.13 (.328) | 96.06 | 89.33 | 89.83 | 2.07 (.132) | **95.95^a^** | **84.76^b^** | **2.49 (.016)** |
| VABS – Soc | 86.61 | 84.03 | 83.92 | 0.46 (.634) | 87.19 | 85.17 | 82.50 | 0.68 (.511) | 87.37 | 80.47 | 1.62 (.112) |
| Groups marked with different superscript letters (a, b) differed significantly with Bonferroni correction applied (*p* <.05).  MSEL-VR: Mullen Scales of Early Learning – Visual reception; ADOS – CSS: Autism Diagnostic Observation Schedule – Calibrated Severity Score; PLS: Preschool Language Scale; VABS: Vineland Adaptive Behavior Scale, Com: Communication, Soc: Socialisation | | | | | | | | | | | |

1. **Discussion**

**4.1 Changes in HR and HRV including calm videos**

In order to look at the increase in HR / decrease in HRV over time and whether this effect is specific to social content or not, we included the calm data (animal videos with classical music) as presented in Bazelmans et al. (2019).


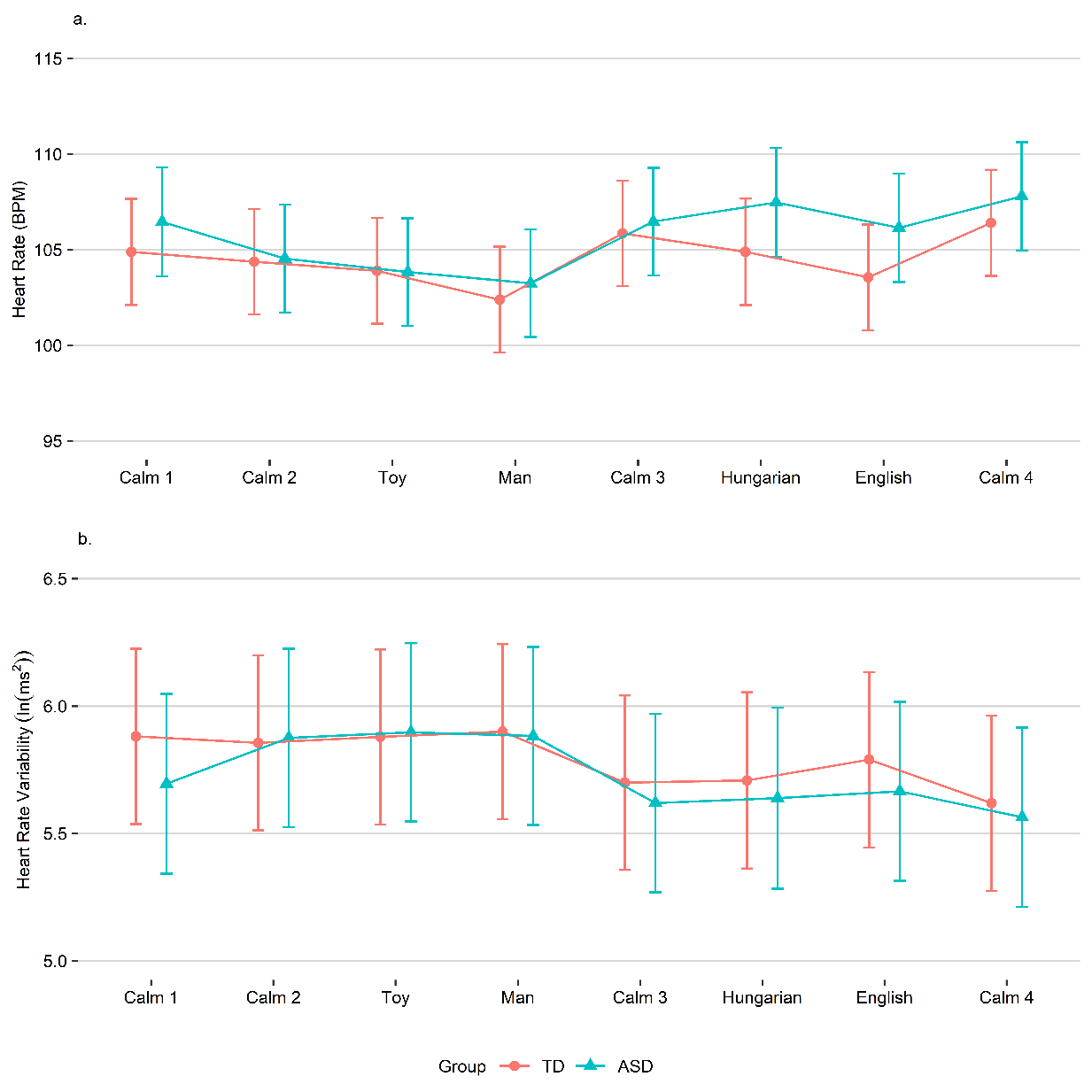


Figure S4. Heart rate and heart rate variability for each condition, including the ‘Calm’ videos as presented in Bazelmans, 2019. All figures are based on the marginal means. Error bars represent 95% confidence interval a. Average heart rate for TD versus ASD, b. Average heart rate variability for TD versus ASD.

References

Bazelmans, T., Jones, E. J. H., Ghods, S., Corrigan, S., Toth, K., Charman, T., et al. (2019). Heart rate mean and variability as a biomarker for phenotypic variation in preschoolers with autism spectrum disorder. *Autism Research, 12*(1), 39-52. doi:10.1002/aur.1982
